# Candidate gene prioritization with Endeavour

**DOI:** 10.1093/nar/gkw365

**Published:** 2016-04-30

**Authors:** Léon-Charles Tranchevent, Amin Ardeshirdavani, Sarah ElShal, Daniel Alcaide, Jan Aerts, Didier Auboeuf, Yves Moreau

**Affiliations:** 1 INSERM U1210, CNRS UMR5239, Laboratoire de Biologie et de Modélisation de la Cellule, Ecole Normale Supérieure de Lyon, Université de Lyon, 69364 Lyon, France; 2 Department of Electrical Engineering (ESAT), STADIUS Center for Dynamical Systems, Signal Processing and Data Analytics Department, KU Leuven, B-3001 Leuven, Belgium; 3 iMinds Future Health Department, KU Leuven, B-3001 Leuven, Belgium

## Abstract

Genomic studies and high-throughput experiments often produce large lists of candidate genes among which only a small fraction are truly relevant to the disease, phenotype or biological process of interest. Gene prioritization tackles this problem by ranking candidate genes by profiling candidates across multiple genomic data sources and integrating this heterogeneous information into a global ranking. We describe an extended version of our gene prioritization method, Endeavour, now available for six species and integrating 75 data sources. The performance (Area Under the Curve) of Endeavour on cross-validation benchmarks using ‘gold standard’ gene sets varies from 88% (for human phenotypes) to 95% (for worm gene function). In addition, we have also validated our approach using a time-stamped benchmark derived from the Human Phenotype Ontology, which provides a setting close to prospective validation. With this benchmark, using 3854 novel gene–phenotype associations, we observe a performance of 82%. Altogether, our results indicate that this extended version of Endeavour efficiently prioritizes candidate genes. The Endeavour web server is freely available at https://endeavour.esat.kuleuven.be/.

## INTRODUCTION

Biologists often use a combination of high-throughput methods, to produce large-scale data and generate hypotheses, and of low-throughput methods, to experimentally validate these hypotheses and create biological knowledge. One challenge in current biology is the gap between the large amount of genomic data that are being generated and the pace at which novel knowledge is created from it. This problem is particularly conspicuous in medical genetics, with many human complex traits and Mendelian disorders remaining unexplained despite the availability of huge amounts of genome-scale data. In this situation, computational biology aims at reducing this gap by proposing *in silico* methods that analyze these data to derive hypotheses that can be validated experimentally.

An example in medical genetics is the identification of the genomic factors underlying human Mendelian disorders. Indeed, high-throughput experiments often produce large lists of candidate genes among which only a few are truly relevant to the disease or phenotype under study. It is not always possible to experimentally validate all candidate genes individually, and so there is the need to prioritize these candidate genes as to maximize the efficiency of the downstream validation experiments. More precisely, the principle is to identify the most promising candidate genes and filter out the ones that appear of limited relevance, and then to investigate these promising candidate genes more thoroughly. The study of complex disorders raises a similar problem since there are many genes involved, each gene having a small effect. The objective is therefore to identify these genes among the numerous candidate genes. Several computational methods have been proposed to tackle this prioritization problem and they have been used in practice to help identify disease-causing genes (1,2).

Some of these methods rely on the guilt-by-association concept as to favor candidate genes that resemble the most what we already know about the disease or phenotype of interest (3,4). For instance, if a candidate gene has a protein product that directly physically interacts with a protein that is already known to be involved in the phenotype of interest, then the candidate gene appears promising for this phenotype. This can be extended by considering multiple sets of data beside protein interaction networks, which is often what prioritization methods do. For instance, the *ToppGene* suite combines an interaction network with functional annotations to favor the candidate genes that interact with the seed genes (which defined the disease of interest) and also have similar functional annotations (5). Similarly, *GeneDistiller* uses gene-phenotype associations, gene expression patterns and protein–protein interactions among other data to propose candidate gene prioritization (6). There exist other methods that do not rely on seed genes, and are therefore more suited for diseases of unknown etiology. These methods rely on disease relevant keywords, or on complementary disease specific experimental data sets. For instance, the *Génie* web server ranks genes using a text-mining approach that is fed with user-selected keywords and can be complemented with orthology information (7). Alternatively, *MetaRanker* integrates multiple disease specific data sets, including genetic association data, copy number variation data and differential expression profiles (8).

In this article, we introduce a much-improved version of *Endeavour*, our gene prioritization method, which is available for six species—now also including zebrafish. Endeavour currently integrates 75 data sources that represent different and complementary views about the genes, which is more than twice as many as our previous version (9). In addition, we have validated Endeavour using a time-stamped benchmark in which predictions are made and only benchmarked later on when enough new data have accumulated (similar to the CAFA strategy (10)).

Other researchers have used Endeavour to decipher the mechanisms underlying several human diseases. For instance, Yu *et al*. used Endeavour to prioritize candidate genes extracted from a whole-exome sequencing of familial cases of congenital diaphragmatic hernia (11). After validation, *GATA4* (ranked 3rd) was associated with congenital diaphragmatic hernia. Zielinski *et al*. studied hemifacial microsomia and identified a 1.3 Mb duplication on chromosome 14q22 by analyzing five affected individuals (12). Their final result suggests a role for *OTX2* (ranked 1st) in human craniofacial development.

## ENDEAVOUR METHODOLOGY

**Figure 1.**
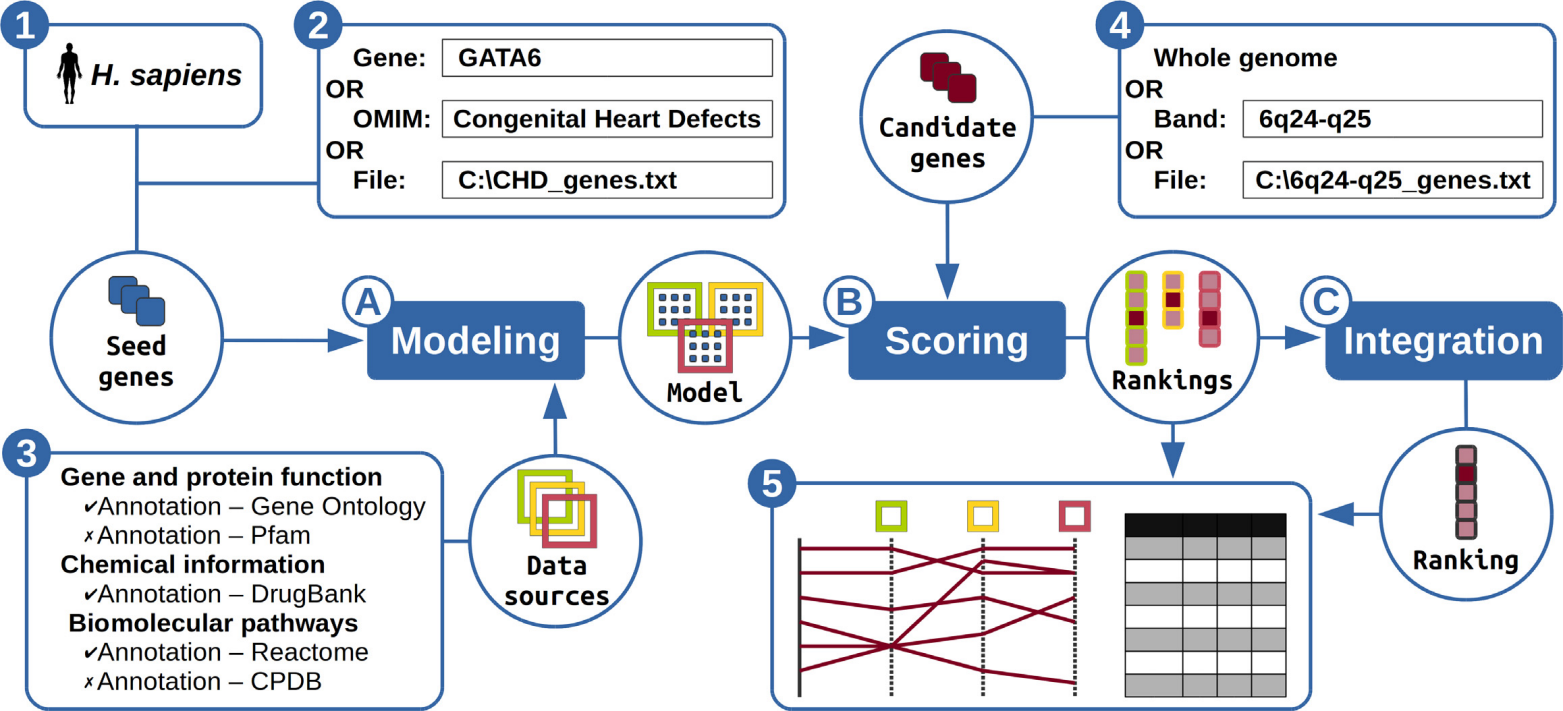
The Endeavour algorithm. Users can start a prioritization by (1) selecting the species of interest, (2) defining which genes are known to be associated with the process of interest, (3) selecting the data sources to be used in the process and (4) providing the candidate genes to prioritize. Endeavour then (**A**) uses the seed genes to build a model of the process of interest, (**B**) scores the candidate genes with this model to produce several rankings and (**C**) integrate these rankings into one global ranking, which (5) is returned to the user through the web server.

Users can prepare a prioritization in four simple stages (see Figure 1). In the first stage, the species with which to work is selected among the six available species (*H. sapiens, M. musculus, R. norvegicus, D. melanogaster, C. elegans* and *D. rerio*). Then, the set of seed genes (or training genes) is prepared in the second stage. These are the genes that are already associated with the process of interest (e.g. genes already associated with congenital heart defects). Typically, users would like to have at least 5 but no more than 40 seed genes, but larger seed sets can still provide reliable results. The third stage consists in selecting the data sources to use for the prioritization. Users are advised to select the resources that best suit their specific problem and to avoid using conjointly redundant resources. The list of usable data sources depends on the selected species. There is no limit in the number of data sources to select, besides its impact on computing time. Finally, the set of candidate genes is defined in the last stage. The hypothesis is that at least one of these candidate genes is associated with the biological process of interest, but this association is yet unknown. This set can for example be derived from previous experiment (e.g. genes from a deletion frequently observed in patients with congenital heart defects). However, there is no size limit regarding this set, so users who have no *a priori* regarding relevant candidate genes can instead prioritize the whole genome. The single output is an ordered list of the candidate genes with on top the most promising candidate genes. In an ideal situation, the gene yet unknown associated with the process of interest should be ranked first or at least among the top ranking genes. Each candidate gene is also given a *P*-value that represents the significance of this combination of rankings. In addition, rankings for each individual data source are also available as to better understand the global ranking (e.g. to identify the sources that contributed the most to prioritize a given gene).

The algorithm behind Endeavour prioritizes genes in three simple steps (see Figure 1). In the first step, it trains a model of the biological process of interest, using the seed genes provided by the user. The model contains one sub-model per user-selected data source and is trained using simple statistics. For instance, for annotation-based data sources (e.g. Gene Ontology) only the features over-represented within the set of seed genes are kept in the sub-model. In the second step, the candidate genes are scored using the model built in the first step. More precisely, for a given data source, the scores are computed using the associated sub-model and represent how well the candidate genes fit this sub-model. For instance, for annotation-based data sources, the score of a candidate gene is the Fisher's omnibus combination of the *P*-values of its annotations that are present in the sub-model (i.e. annotations not present in the sub-model are ignored). At the end of this step, each data source is associated with a ranking of the candidate genes, with the most promising candidate genes on top. In the third step, these rankings, which correspond to prioritizations made using different data sources, are then integrated using order statistics to obtain a single global ranking (13). This method allows candidate genes with little data (e.g. poorly annotated genes) to be fairly compared with candidate genes that have a lot more data. The algorithm then outputs a ranked list of candidate genes, with *P*-values that represent the significance of this ranking. In addition, the prioritization results are now displayed graphically using a parallel coordinate representation so that users can easily check which data sources contributed the most to the global ranking.

Endeavour integrates 75 data sources for six species that can be classified into broad categories that describe what we know about genes. They are briefly described below, and a detailed list of all available databases is available in Supplementary Material 1. The ‘Gene and protein function’ category includes resources such as Gene Ontology (14) and InterPro (15), which are usually organized as annotations between ontologies (that describe function in a broad sense) and biological entities (i.e. genes or gene products). In addition, the category ‘Biomolecular pathways’ includes pathway databases such as Reactome (16), which are complementary to the purely ontological annotations described above. Several resources that describe gene or protein interaction networks, such as BioGrid (17) and IntAct (18), are also integrated and classified into the ‘Interaction networks’ category. Chemical data sets are also integrated in Endeavour within the ‘Chemical information’ category. These contain annotations between gene or gene products and other entities such as drugs. One example is the DrugBank database (19). The ‘Phenotypic information’ category gathers all resources that collect associations between genes or gene products and phenotypes or diseases, possibly using model organism data. Examples are the OMIM database for human Mendelian disorders (20) and the Rat Disease Ontology from the Rat Genome Database (21). Expression data are split into two categories depending on whether complete expression profiles are available (category ‘Expression profiles’), such as large expression data sets, or whether the data have already been summarized into annotations, such as within PaGenBase (22) (category ‘Expression ontologies’). The last category ‘Sequence-based features’ contains all resources that are based on gene, transcript or protein sequences. This includes protein sequence similarities computed using BLAST or predicted miRNA regulation using transcript sequences (23,24).

The web server is based on a three-tier architecture, developed using Microsoft .Net technology and Microsoft SQL database. The core Endeavour library is written in Perl and the data are stored in a MySQL database. We have introduced a parallel coordinate representation to visualize our multidimensional numerical results. This gives the users the opportunity to easily recognize patterns in the data and identify possible correlation among the different sources. Uploaded data and prioritization results are kept private and not viewable by other users, and are in any case deleted after 30 days. The web site is free and open to all and there is no login requirement. Two examples derived from the literature are available for users who simply want to try out Endeavour. In addition, a manual describes how to run a simple prioritization and a help page contains hints on how to solve the more frequent issues.

## EVALUATION RESULTS

For the first validation, we have performed a leave-one-out cross-validation on ‘gold standard’ gene sets. These sets contain genes already associated with phenotypes or biological processes and have been extracted from reference databases, such as OMIM and Gene Ontology. These sets are mostly derived from studies on Mendelian disorders that represent the majority of our current knowledge. However, we have also included a benchmark data set extracted from the Genetic Association Database that focuses on complex disorders, since these are the focus of most of the studies nowadays. Briefly, for each iteration of the cross-validation, the prioritization is done by training Endeavour with all genes from the ‘gold standard’ set except one (hereafter called the left-out gene), and the aim is to rank this left-out gene together with 99 randomly selected genes. We expect this left-out gene to rank toward the top since it comes from the ‘gold standard’ set. All data sources except the ones that contain explicitly the association we want to predict are used in the process (i.e. OMIM is not used for any phenotype-based ‘gold standard’). In the end, an Area Under the ROC curve (AUC) is computed for each ‘gold standard’ set, and AUC values are averaged over the different sets to derive a global estimate. These are then compared to control AUC values obtained with randomly built gene sets. A summary of the results is presented in Table 1. The full results, including ROC curves, are available in Supplementary Materials 2 and 3. We observe that performance varies from 88.34% (for human phenotypes) to 94.93% (for worm gene function), which indicates that Endeavour methodology is performing as expected. In addition, our benchmark reveals that the performance on complex disorders (GAD, 88.96%) is similar to the performance on pure Mendelian disorders (OMIM, 93.41%). Based on the GAD benchmark, we also observe that the average running time for the prioritization of 100 human genes is 311 s (using 34 data sources).

**Table 1.** Results of the leave-one-out cross-validation on ‘gold standard’ gene sets

Species	Source	Nb sets	Nb genes	AUC	Control AUC
*H. sapiens*	HPO	1553	19 386	88.34%	49.79%
	OMIM	29	611	93.41%	48.43%
	GAD	966	10 921	88.96%	50.04%
	GO	4526	55 930	92.26%	49.93%
*M. musculus*	RGD-RDO	672	8413	88.61%	49.59%
	GO	4 379	53 105	90.46%	49.75%
*R. norvegicus*	RGD-RDO	652	7997	90.55%	49.21%
	GO	4140	49 895	88.68%	49.24%
*D. melanogaster*	FlyBase-Pheno	1612	17 395	91.38%	50.12%
	GO	2371	28 834	89.88%	49.93%
*C. elegans*	WormBase-Func	225	2304	94.93%	50.87%
	GO	1400	17 060	92.05%	49.95%
*D. rerio*	Zfin-Pato	135	1662	88.70%	49.37%
	GO	1856	22 476	90.76%	49.16%

For each benchmark (row), the columns contain the number of gene sets, the number of genes, the AUC and the control AUC respectively.

However, cross-validation is often not the best method to estimate actual performance (because leakage of information across multiple sources can lead to overly optimistic results), and time-stamped strategies are often preferable (25). We have therefore defined a time-stamped benchmark that resembles the CAFA strategy (10). The principle is that predictions have been made in January 2013 and only benchmarked in December 2015 to avoid data contamination (i.e. data used for both training and testing, even if indirectly). Our benchmark relies on annotations between human genes and phenotypic terms from the Human Phenotype Ontology (HPO). More precisely, Endeavour was first used to make predictions for a wide range of HPO terms, using the whole genome and only data from January 2013 or before. We have then collected from HPO the 3854 phenotype-gene associations that have been discovered between January 2013 (build 44) and December 2015 (build 102), and used these associations to benchmark the Endeavour predictions from the first step. The AUC over the 3854 novel associations is 81.51%, which indicates that Endeavour is able to efficiently prioritize disease candidate genes in a realistic setup (details are in Supplementary Material 4). However, this AUC value is lower than the estimate obtained by the cross-validation, therefore confirming the suspicion that cross-validation on a ‘gold standard’ most likely provides an overestimation of the actual performance.

## CONCLUSION

Endeavour is a web server that performs gene prioritization for six species (*H. sapiens, M. musculus, R. norvegicus, D. melanogaster, C. elegans* and *D. rerio*). It is available through a web-based user interface. Endeavour has been validated using a standard cross-validation scheme, as well as a time-stamped benchmark. Altogether, we provide a server that supports researchers in deciphering the molecular basis underlying diseases and phenotypes.

## Supplementary Material

SUPPLEMENTARY DATA
