# Supplementary material for: Candidate gene prioritization with Endeavour
Source: Nucleic Acids Res. 2016 Apr 30;44(Web Server issue):W117–21. doi: 10.1093/nar/gkw365 (PMC4987917; doi:10.1093/nar/gkw365)
Supplement: SUPPLEMENTARY DATA [file supp_44_W1_W117__index.html]

Candidate gene prioritization with Endeavour — SUPPLEMENTARY DATA 

# Candidate gene prioritization with Endeavour

## SUPPLEMENTARY DATA

- SUPPLEMENTARY DATA
- SUPPLEMENTARY DATA
- SUPPLEMENTARY DATA
- SUPPLEMENTARY DATA
