# Supplementary material for: Candidate gene prioritization with Endeavour
Source: Nucleic Acids Res. 2016 Apr 30;44(Web Server issue):W117–21. doi: 10.1093/nar/gkw365 (PMC4987917; doi:10.1093/nar/gkw365)
Supplement: SUPPLEMENTARY DATA [file supp_gkw365_nar-00568-web-b-2016-File006.pdf]

Candidate gene prioritization with Endeavour: supplementary figures

Léon-Charles Tranchevent, Amin Ardeshirdavani, Sarah ElShal, Daniel Alcaide, Jan Aerts, Didier Auboeuf, and Yves Moreau

April 11, 2016

These figures represent Receiver Operating Characteristics (ROC) curves derived from the Endeavour benchmarks. Each figures contains two curves, a first one for the real benchmark (using golden standard data sets), and a second one for the control experiments (using randomly generated data sets of the same sizes). In addition, the Area Under the Curve (AUC) is indicated in both cases as an estimate of the performance.

Abbreviations used:

GAD
Genetic Association Database

GO
Gene Ontology

HPO
Human Phenotype Ontology

OMIM
Online Mendelian Inheritance in Man

PATO
Phenotypic Attribute Trait Ontology

RDO
Rat Disease Ontology

RGD
Rat Genome Database

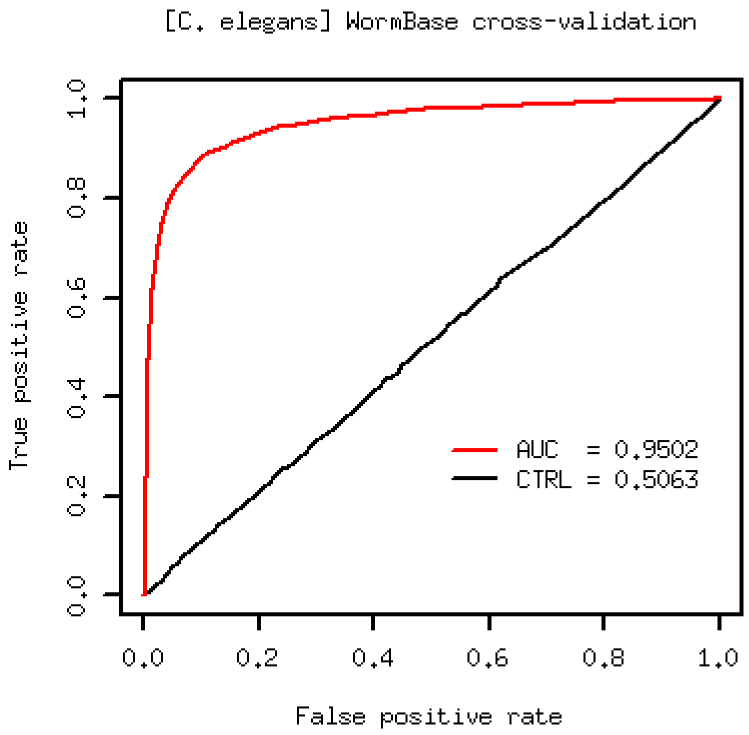

Figure 1: ROC curve for the *C. elegans* WormBase function based benchmark.

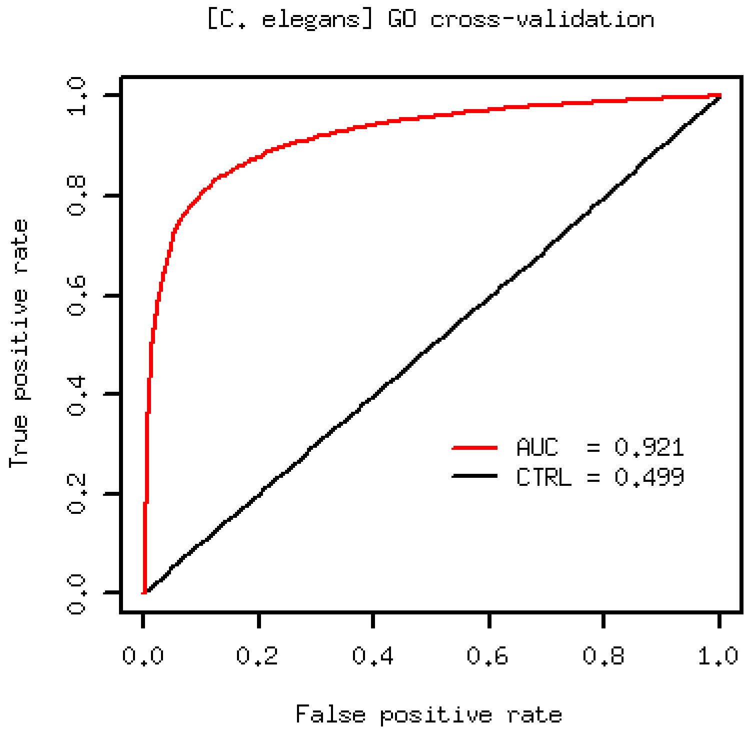

Figure 2: ROC curve for the *C. elegans* GO based benchmark.

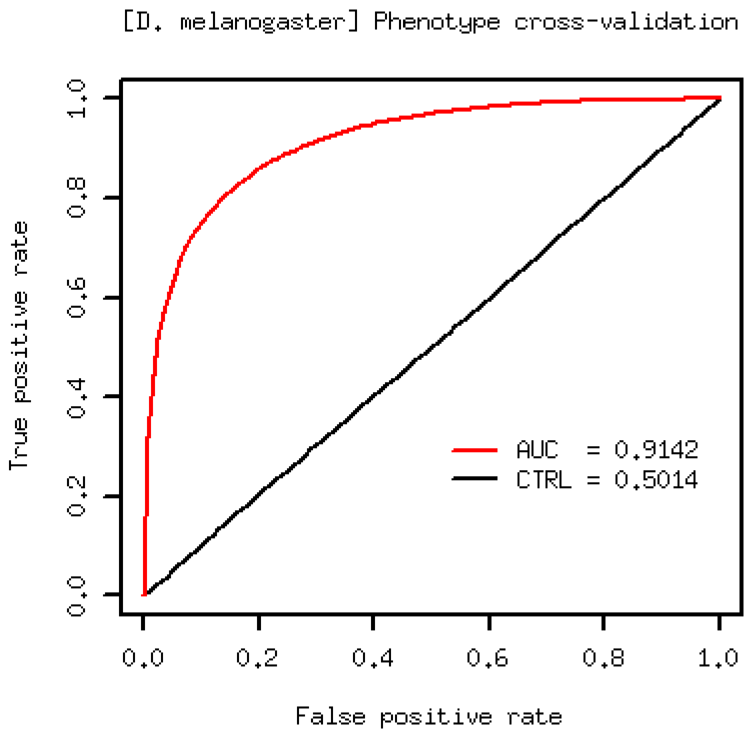

Figure 3: ROC curve for the *D. melanogaster* FlyBase phenotype based benchmark.

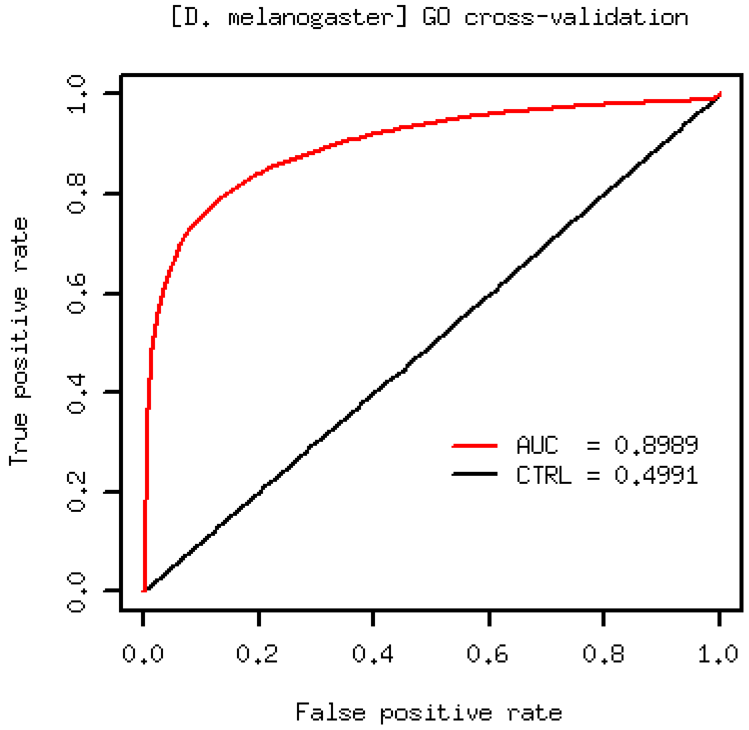

Figure 4: ROC curve for the *D. melanogaster* GO based benchmark.

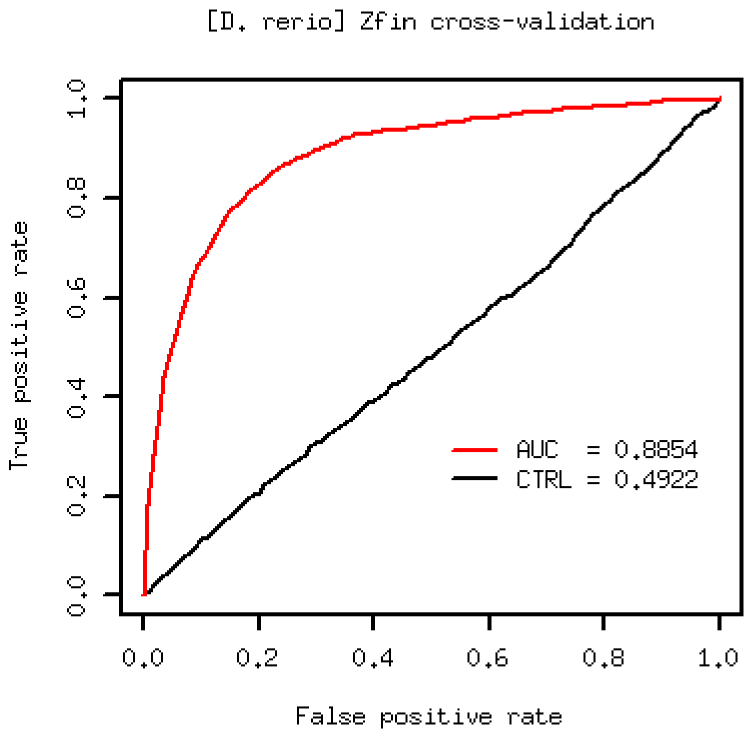

Figure 5: ROC curve for the *D. rerio* Zfin PATO based benchmark.

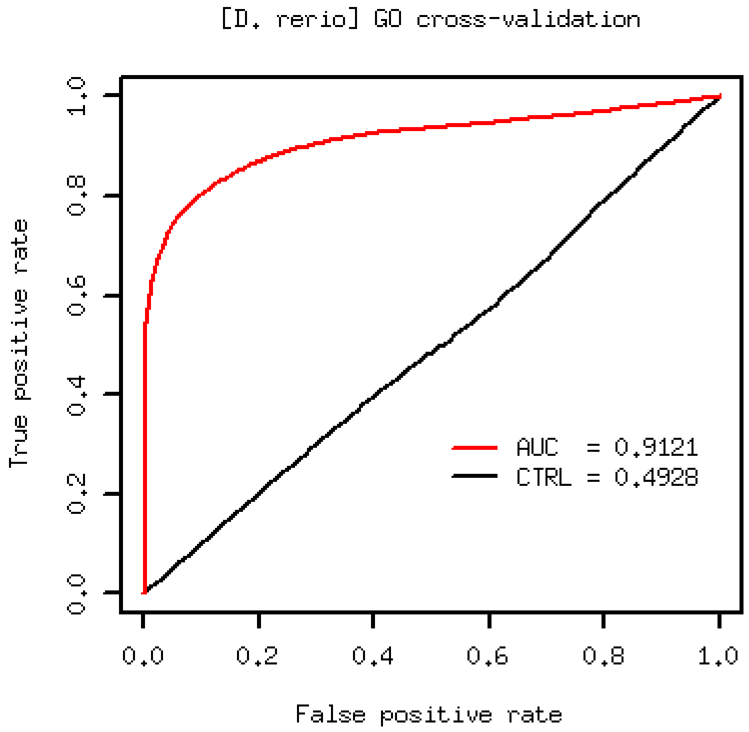

Figure 6: ROC curve for the *D. rerio* GO based benchmark.

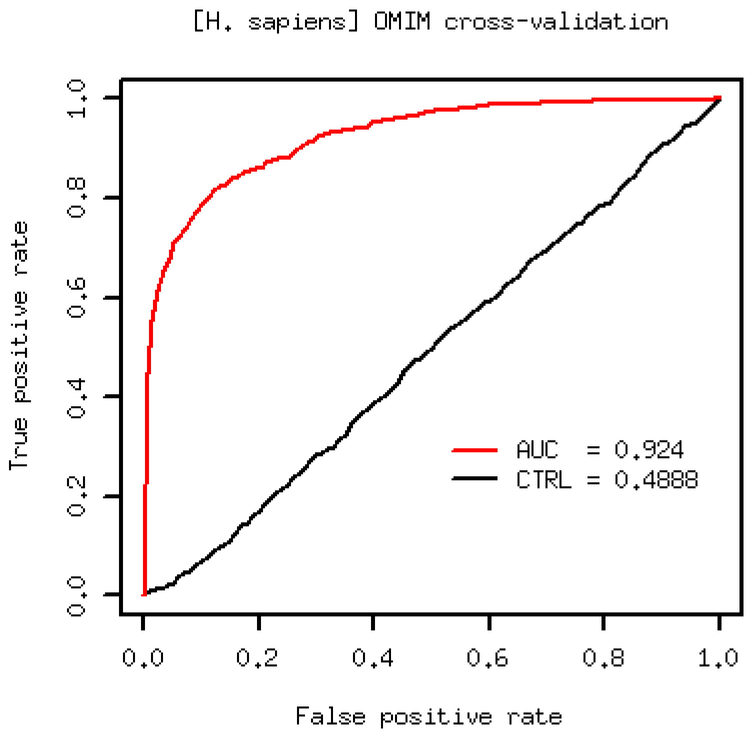

Figure 7: ROC curve for the *H. sapiens* OMIM based benchmark.

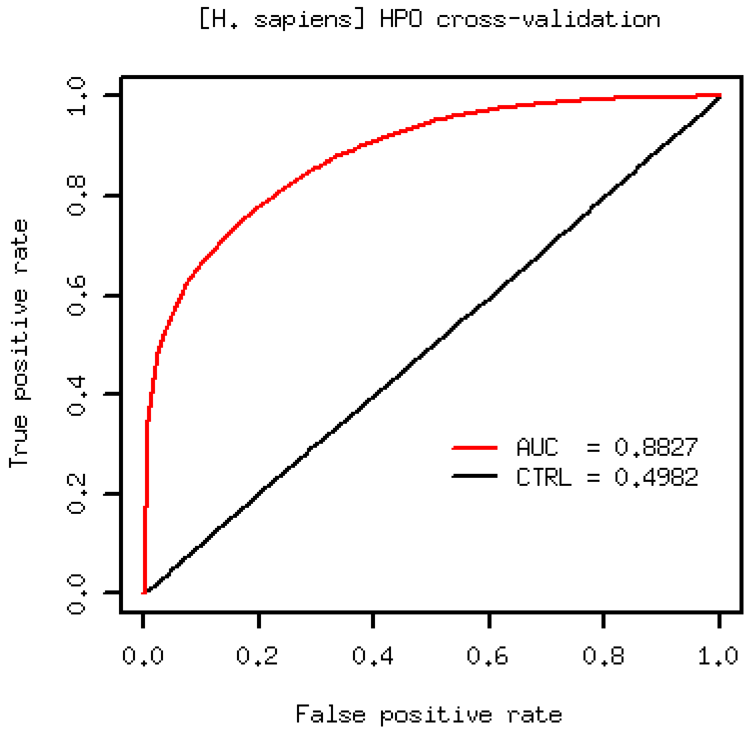

Figure 8: ROC curve for the *H. sapiens* HPO based benchmark.

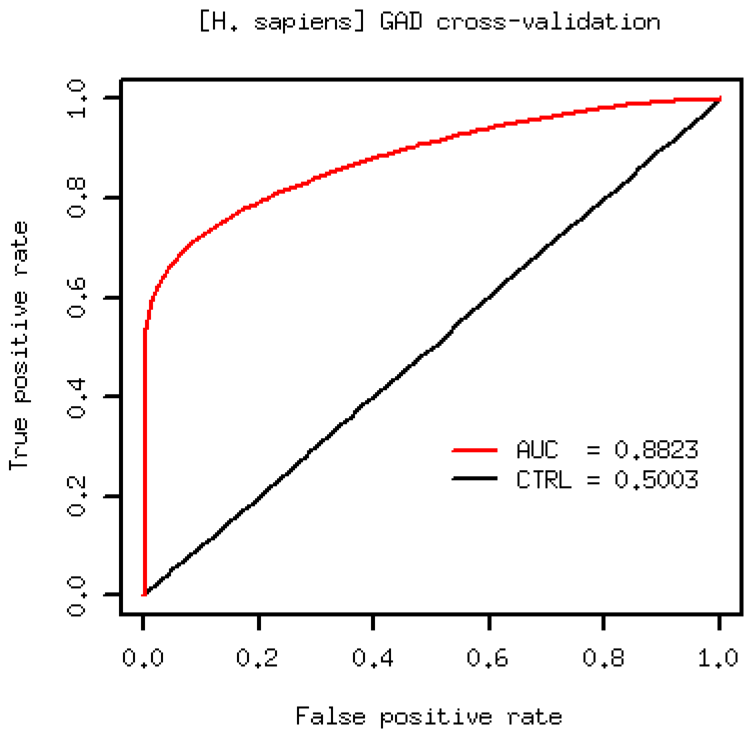

Figure 9: ROC curve for the *H. sapiens* GAD based benchmark.

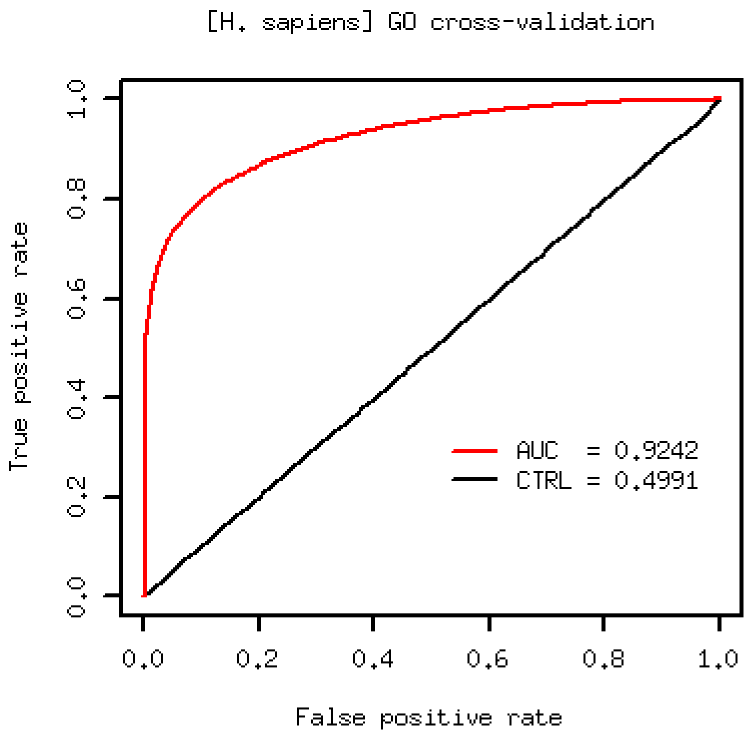

Figure 10: ROC curve for the *H. sapiens* GO based benchmark.

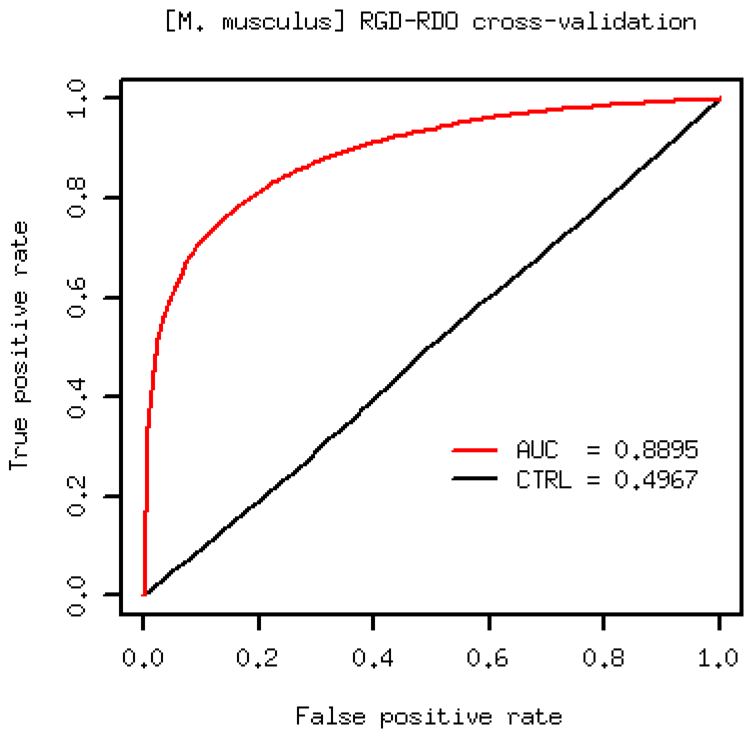

Figure 11: ROC curve for the *M. musculus* RGD RDO based benchmark.

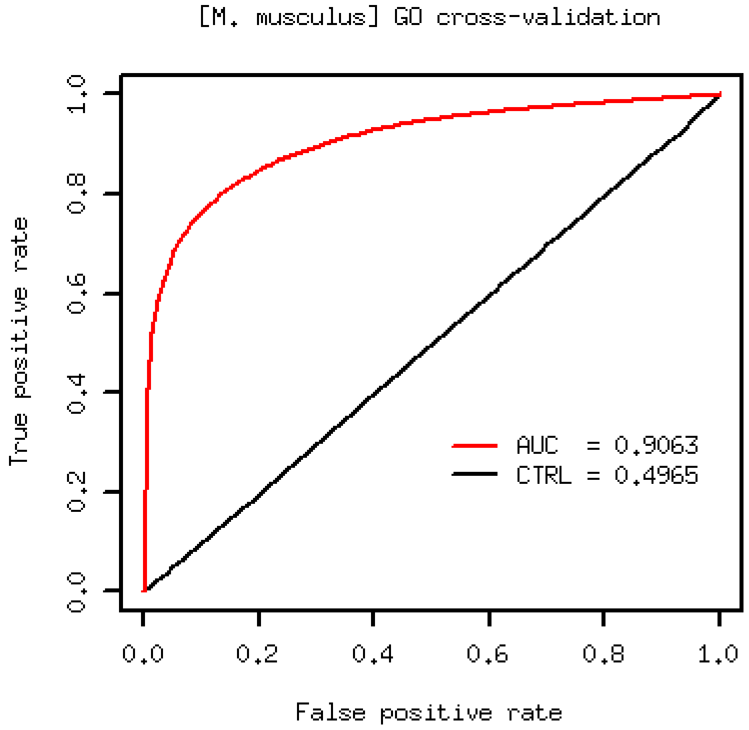

Figure 12: ROC curve for the *M. musculus* GO based benchmark.

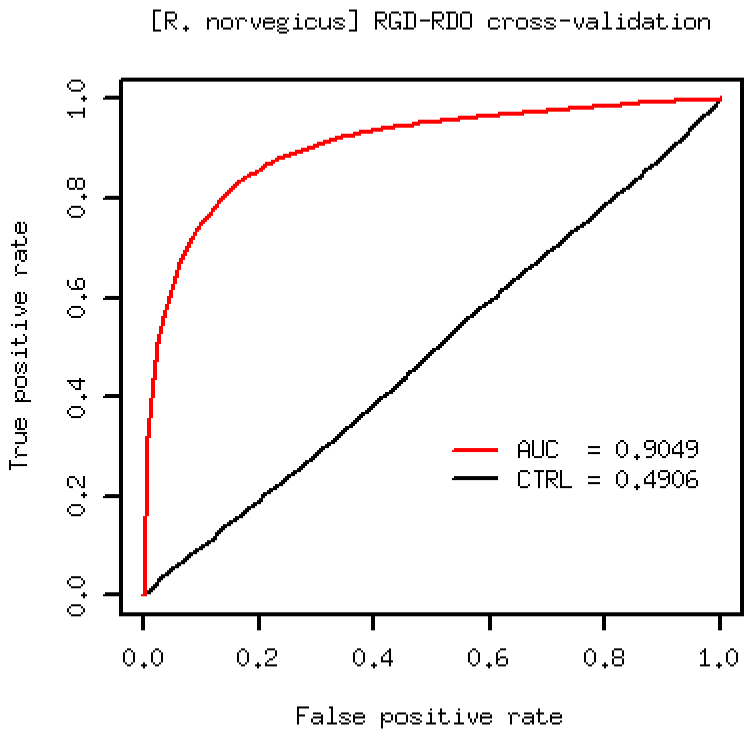

Figure 13: ROC curve for the *R. norvegicus* RGD RDO based benchmark.

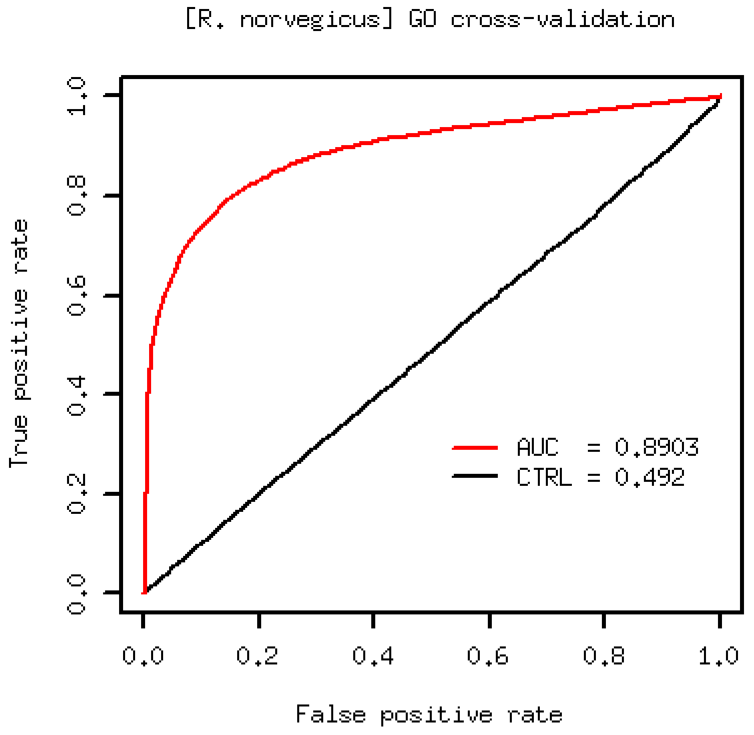

Figure 14: ROC curve for the *R. norvegicus* GO based benchmark.
